# The efficacy of acupuncture for the treatment and the fertility improvement in child-bearing period female with Hashimoto Disease: A randomized controlled study

**DOI:** 10.1097/MD.0000000000020909

**Published:** 2020-07-02

**Authors:** Fangyuan Li, Zhang Qi, Lu Hua, Xinxin Wang, Mi Ling, Du Juan

**Affiliations:** a College of Clinical Medicine, Chengdu University of Traditional Chinese Medicine; b Chengdu University of Traditional Chinese Medicine Affiliated Hospital; c College of Acupuncture and Tuina, Chengdu University of Traditional Chinese Medicine, Chengdu; d Maternal and Child Reproductive Hospital affiliated to Chengdu University of Traditional Chinese Medicine, Chengdu, Sichuan Province, PR China.

**Keywords:** acupuncture, effectiveness, fertility, Hashimoto thyroiditis, RCT

## Abstract

**Background:**

Hashimoto thyroiditis (HT) is highly prevalent among reproductive-aged women and has a substantial negative impact on fertility. Currently, there is no specific treatment for Hashimoto thyroiditis. We hypothesize that acupuncture can halt or delay the progression of HT and improve fertility in child-bearing period female. We therefore designed a randomized controlled trial to test this hypothesis by comparing the therapeutic effect of acupuncture vs sham acupuncture in patients with Hashimoto thyroiditis.

**Methods:**

In this randomized controlled study, a total of 284 eligible patients will be assigned to acupuncture group (n = 142) or sham acupuncture group (n = 142) in a 1:1 ratio. All patients will receive 36 sessions in total for 12 consecutive weeks with the same acupoint prescription (RN23, ST9, RN17, RN4, RN6, ST36, SP6, KI6). The primary assessment is the titers of thyroid peroxidase antibodies (TPOAb) and thyroglobulin antibody (TGAb). Secondary outcomes include the thyroid function, ovarian function, the rate of primary ovarian insufficiency, and pregnancy outcome. The thyroid function and thyroid antibodies tests will be measured at weeks 0, 4, 8, and 12 after randomization. The ovarian function will be examined on the 2nd to 4th day of the menstrual period in the 1st month, 2nd month and 3rd month compared with baseline. Both the pregnancy outcome and the rate of primary ovarian insufficiency will be evaluated 1 year after treatment.

**Discussion:**

This will be the first large-scale trial specifically evaluating acupuncture therapy in child-bearing period female with Hashimoto thyroiditis. If the study confirms the effectiveness of acupuncture treatment, more consistent acupuncture therapy can be set up for clinical practice.

**Trial registration:**

Chinese Clinical Trials Register identifier, ChiCTR2000031320, registered on 27 March 2020.

## 1 Introduction

Hashimoto thyroiditis (HT) known as autoimmune thyroiditis is a common autoimmune thyroid disease (AITD) and endocrine disorder.^[1–3]^ Surveys have shown that the prevalence of thyroid autoimmunity(TAI) and HT is 6% to 20%^[4]^ and 5% to 15%^[5]^ among reproductive-aged women, respectively. HT is characterized by increased levels of thyroid peroxidase antibodies (TPOAb) and thyroglobulin antibody (TGAb), diffuse lymphocytic infiltration of the thyroid gland, manifestations of goitrous or atrophic gland, and frequent thyroid dysfunction in varying degrees.^[6,7]^ HT is the most common cause of hypothyroidism.^[8]^ The pathogenesis is thought to be an immunologic attack that the presence of TPOAb tends to correlate with thyroidal damage and lymphocytic inflammation.^[9]^ TPOAb is directly cytotoxic to thyrocytes.^[10]^ In patients who have circulating thyroid peroxidase antibodies, there is a greater risk of progression from subclinical to overt hypothyroidism.^[11]^ A previous study indicates that largely increased levels of TPOAb are associated with a moderately increased risk of developing hypothyroidism in the patients of Hashimoto thyroiditis remained euthyroid.^[11]^ An epidemiological survey with a 20-year follow-up showed that the lifetime risk of incident hypothyroidism was 4% among TPOAb negative women, 23% for women with low-level TPOAb, 33% for those with mid-level TPOAb, and 53% for those with high TPOAb.^[12]^ Most cases of Hashimoto thyroiditis are considered to be the early stage of hypothyroidism. According to studies, 4% to 8% of the reproductive-age population suffered from subclinical hypothyroidism (SCH) with elevated thyroid stimulating hormone (TSH) more than 4.5 to 5.0 mIU/L and normal free thyroxine (FT4) levels concentration.^[13–15]^ The risk of progression from SCH to overt hypothyroidism (OH) ranges between 2% and 5% a year.^[12]^ It is well known that thyroid hormones play important roles during the development and maintenance of reproductive function in women.^[16]^ Numerous studies have suggested that thyroid autoimmunity and hypothyroidism associated with an adverse effect on fertility as well as pregnancy.^[4,17–21]^

For two decades, the association between TAI and reproductive failure has gained attention.^[17,22]^ Fertility is impaired in women with autoimmune thyroid disease.^[23]^ An extensive meta-analysis study reported that the presence of TPOAb combined with normal thyroid function was associated with an increased risk of unexplained subfertility, implantation failure, miscarriage, recurrent miscarriage, preterm birth, and maternal post-partum thyroiditis.^[24,25]^ A prospective study confirmed that the prevalence of TPOAb was greater (18%) in infertile women than that in healthy and fertile controls (8%).^[26]^ Autoimmune thyroid disorder and hypothyroidism have a negative impact on ovarian function.^[27,28]^ Women with AITD have lower serum anti-Müllerian hormone (AMH) levels compared with age-matched controls, which means women with AITD have prematurely aging ovaries.^[29]^ A prospective controlled trial revealed that 5% of patients of premature ovarian insufficiency (POI) presented highly elevated antithyroid antibodies.^[30]^ Besides, 24.1% of patients with premature ovarian failure (POF) had thyroid peroxidase autoantibodies.^[31]^ Idiopathic low ovarian reserve was associated with more frequent positive TPOAb in Chinese women.^[32]^ The human ovary is often the target of an autoimmune attack.^[33]^ In this context, HT is likely to be associated with ovarian dysfunction and diminished ovarian reserve. TPOAb has been detected in all samples of follicular fluid obtained from women with thyroid autoimmunity, while they were absent in women without thyroid autoimmunity. In these patients with HT, the levels of TPOAb in ovarian follicular fluid is positively correlated with serum antibody levels.^[34]^ Monteleone et al speculated that TPOAb might passed through the blood follicle barrier during follicular evolution and damage growing follicles and oocytes via thyroid hormone receptors on granulosa cells, which may result in decreasing the quality and development potential of oocyte.^[34]^ TPOAb targets TPO antigen on granulosa cells, creating a hostile local immune microenvironment around the oocyte.^[18]^ Subclinical or overt hypothyroidism is often caused by HT, which is associated with a diminished ovarian reserve in infertile patients of reproductive age.^[35,36]^ Recently, a retrospective study showed that SCH was associated with decreased ovarian reserve during later reproductive age.^[37]^ Therefore, Hashimoto thyroiditis has a substantial negative impact on the fecundity of reproductive-aged women. Impaired fertility has become a major public health problem that is associated with an increasing burden on society.

To date, there are no specific therapies to effectively reduce the autoantibody level of HT. Patients with HT who have normal thyroid function and no obvious symptoms of oppression should be followed up and monitored thyroid function regularly.^[38]^ Current therapeutic strategies for HT with normal thyroid function in the early stages include Se supplementation, glucocorticoids, and vitamin D.^[39–41]^ However, the available data on beneficial effects of Se on thyroid autoimmune parameters are limited.^[42]^ On the other hand, caution should be taken when long-term selenium supplementation, the increased risk of type 2 diabetes was observed in a randomized clinical trial.^[43]^ Only an ambiguous causal relationship and few interventional studies reported that Vitamin D is beneficial in the management of thyroid disease, so the therapeutic potential of vitamin D remains debated.^[41]^ Although some small trials appear promising of glucocorticoids, corticosteroid use has many negative effects on key aspects of early pregnancy.^[44,45]^ Once overt hypothyroidism is present, levothyroxine (LT4) is the treatment of choice for Hashimoto thyroiditis.^[9]^ Patients with HT who have the mere presence of detectable TPO antibodies does not, however, advocate empiric treatment with thyroid hormone.^[46]^ According to one study, women that are considered euthyroid, before pregnancy, are reclassified as having subclinical hypothyroidism, in cases of TSH between 2.5 and 4 mIU/L.^[47]^ Most endocrinologists use a TSH value of 2.5 mIU/L as the threshold for diagnosing hypothyroidism and starting levothyroxine in pregnant women because of an evident association between mild thyroid impairment and adverse outcomes in pregnancy.^[48]^ LT4 supplementation can slow the progression of HT, control the early stage of hypothyroidism, and support follicular development.^[49]^ The American Thyroid Association's recommendations for LT4 treatment also be considered for TPOAb positive women with normal thyroid function, although the quality of evidence is low.^[50]^ However, LT4 treatment before pregnancy in women is lacking sufficient data. An ideal TSH value before pregnancy can initiate a typical starting dose LT4 therapy (25–50 ug/d) When considering the potential benefits and risks minimization.^[50]^

According to the World Health Organization (WHO), acupuncture can be used to treat thyroid diseases. As integrative therapy or alternative medicine, acupuncture is safe and economical. Acupuncture can reduce symptoms and improve relevant biomarkers of thyroid disease patients.^[51]^ MOK pharmacopuncture ( a new form of acupuncture treatment) can regulate the imbalance of Th1/Th2 cytokines and help to suppress autoimmune response.^[52]^ Acupuncture can regulate the indexes related to immune function and correct the immune dysfunction to some extent, which is possibly the action mechanism of acupuncture-moxibustion in treating HT.^[53]^ Electroacupuncture (EA) intervention can regulate the thyroid hormone.^[54]^ The connection between thyroid and fertility is mainly based on cross-talk between the hypothalamic–pituitary–thyroid (HPT) axis and the hypothalamic–pituitary–gonadal (HPG) axis.^[16]^ The electroacupuncture has a benign regulating effect on the key hormones in the pituitary-target gland axis, such as thyroid-stimulating hormone (TSH), triiodothyronine (T3), tetraiodothyronine (T4), estradiol (E2), luteinizing hormone (LH) and follicle-stimulating hormone (FSH).^[55]^ Therefore, we can draw the preliminary conclusion that acupuncture can delay the progress of Hashimoto's thyroiditis and improve its fertility. Although acupuncture is increasingly used as a nonpharmacologic therapy in clinical practice, the level of available evidence is rated as “low”. We designed an RCT to evaluate the effects of acupuncture on halting or delaying the progression of HT and fertility improving in child-bearing period female.

## 2 Methods and analysis

### 2.1 Design

**Figure 1:**
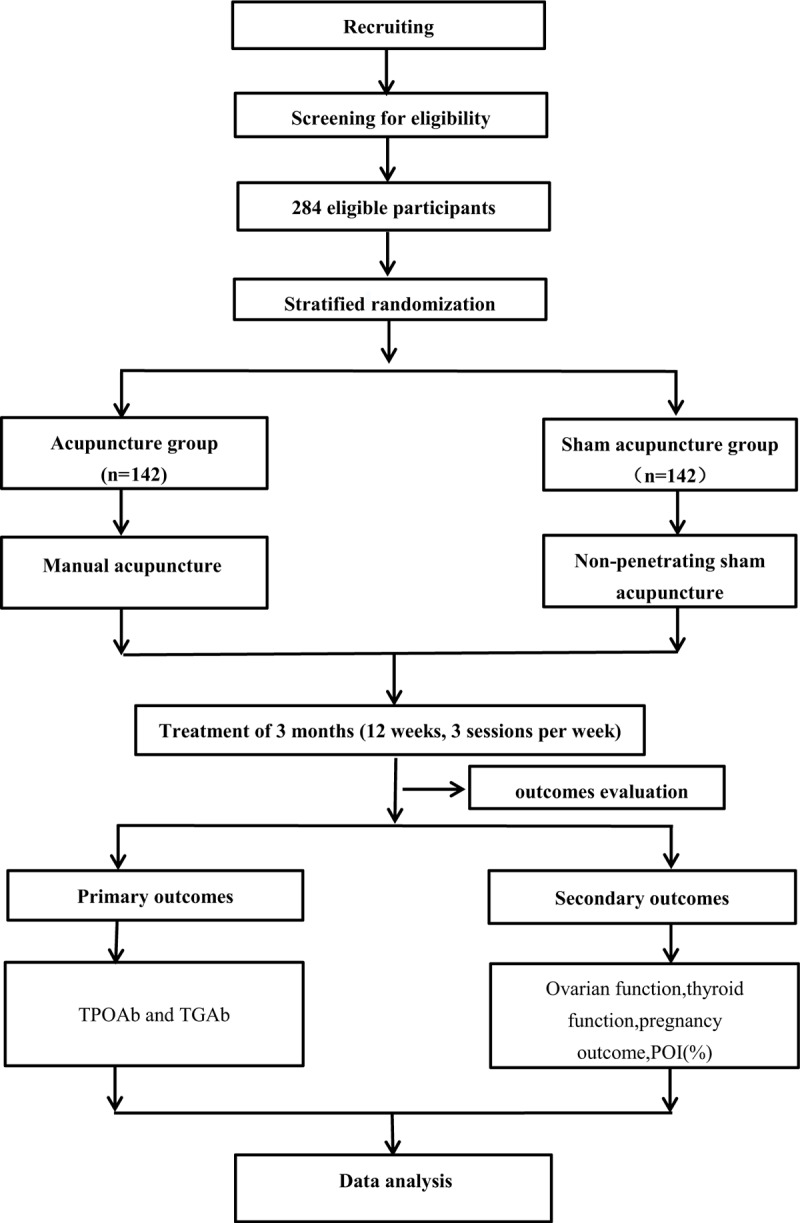
Trial flow chart.

A single-center, patient-blinded, randomized controlled trial (RCT) is currently being performed. Acupuncture will be compared with sham acupuncture to determine if acupuncture has an effect on the reduction of antibody (TPOAb or TGAb) and fertility improving. The trial will be conducted in the Department of gynecology, Teaching Hospital of Chengdu University of Traditional Chinese Medicine. The protocol will be reported following the Consolidated Standards of Reporting Trials (CONSORT) statement and the Revised Standards for Reporting Interventions in Clinical Trials of Acupuncture (STRICTA).^[56,57]^ The flowchart of the trial is shown in Figure 1. The study schedule is detailed in Table 1. The Standard Protocol Items: Recommendations for Interventional Trials (SPIRIT) checklist is provided as Additional File 1.

**Table 1:**
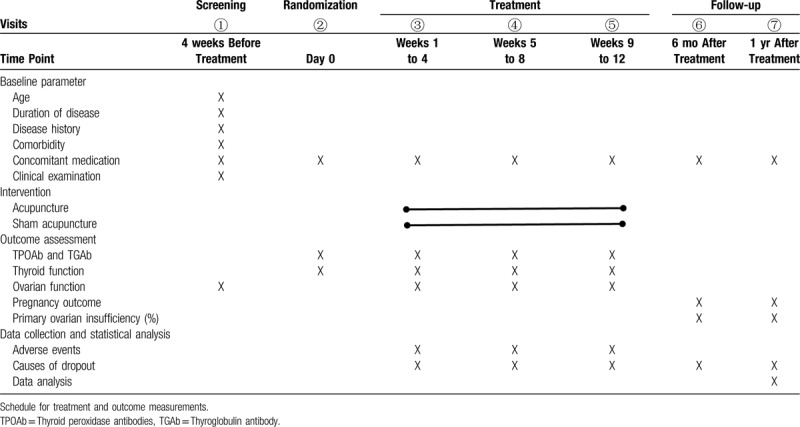
Data collection points.

### 2.2 Ethic approval

The research protocol is performed in accordance with the principles of the Declaration of Helsinki^[58]^ and has been approved by the Sichuan Regional Ethics Review Committee on Traditional Chinese Medicine (approval no. 2019KL-072). We registered the study on the Chinese Clinical Trial Registry (Registration No. ChiCTR2000031320).

### 2.3 Participants

A total of 284 women with HT in child-bearing period will be enrolled in this study after their informed consents are obtained. The diagnosis of HT based on circulating antibodies to thyroperoxidase (mainly thyroperoxidase and thyroglobulin) and reduced echogenicity on thyroid sonogram.^[7]^ If they meet the study criteria, they will be invited to the Department of gynecology, Teaching Hospital of Chengdu University of Traditional Chinese Medicine to undergo the study. All participants will be recruited through hospital social media (WeChat) or posters in the community and hospitals. If a patient is interested in participating, she will contact the researcher by WeChat or telephone. Prospective participants will be asked to talk face to face with the researcher for a baseline screening visit after diagnosis. Eligible participants will be randomized to acupuncture group or sham acupuncture group. There will be one year follow-up period after treatment.

### 2.4 Inclusion criteria

Patients who meet all of the following conditions will be considered for enrollment. The inclusion criteria are as follows:

1. females, aged between 20 and 45 years;
2. meet the diagnosis of HT with normal thyroid function(TSH<5.0 mIU/L) and is trying to conceive;
3. willing to join this research and sign an informed consent form.

### 2.5 Exclusion criteria

The exclusion criteria are as follows:

1. infertility caused by organic lesions of the reproductive system or male infertility;
2. pregnant women or women in their lactation period;
3. complicated with bleeding disorders (for example, thrombocytopenia with bleeding tendency, coagulation disorder), mental diseases (serious anxiety and depression, schizophrenia), malignant tumor, or serious organic diseases;
4. history of radioiodine therapy or surgical intervention of the thyroid;
5. use of immunosuppressants, immunostimulants, or drugs that interfere with the production, transport, and metabolism of thyroid hormones (e.g., corticosteroids, lithium, and amiodarone);
6. participated in other clinical trials in the past 3 months.

### 2.6 Sample size

Sample size will be decided by the primary outcome. The primary outcome of this study will be the mean change of TPOAb and TGAb from baseline to week 12. According to the results of our previous pilot study, titers of TPOAb was 253.86 ± 178.23 IU/ml in the acupuncture group and 318.98 ± 201.37IU/ml in the sham acupuncture group (TPOAb higher than 34IU/ml was the positive threshold of this research center). Considering participants recruited in the two groups are in a 1:1 ratio, the following formula was used to estimate sample size: 
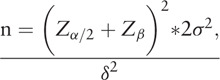


With a 5% significance level (*α* = 0.05, two-sided) and 80% power (*β* = 0.2), at least 134 patients should be enrolled in each group and 284 total participants will be recruited to allow for a 10% dropout rate.

### 2.7 Randomization

As the cutoff value of LT4 supplementation based on TSH level is controversial in women who are trying to conceive, the participants will be stratified by TSH level (TSH < 2.5 mIU/L vs 2.5 mIU/L≤TSH < 5 mIU/L). Once consent has been obtained and baseline data collected, participants will be randomized to the acupuncture group (n = 142) or the the sham acupuncture group (n = 142). Randomization will be performed according to a random list of numbers generated with SPSS21.0 software (International Business Machines Corp., Armonk, NY). An independent researcher will prepare the assignments in opaque envelopes containing an allocation sequence number and take charge of the allocation sequence concealment.

### 2.8 Blinding

Due to the specific nature of the intervention, blinding will be maintained for participants, the data collection researcher, and the statistician except for the acupuncturist. The acupuncturist, the data collection researcher, and data analysts are not allowed to exchange information.

## 3 Interventions

There will be 4 weeks for baseline, 12 weeks for treatment, and 1 year for follow-up in this study. Each participant will receive 36 sessions of 30-minute duration over 12 weeks (3 times per week, once every 2–3 days). Participants whose TSH is greater than 2.5mIU/L should be managed with a typical starting dose LT4 (25 ug/d). TSH levels should be optimized to <2.5 mIU/l, before conception. No additional treatment is allowed during treatment. Only licensed acupuncturists who have more than 5 years of experience will perform the treatment following the standardized operating procedures. The two groups of participants will be treated separately in different rooms to avoid communication between the participants. Each participant will take a supine position on the treatment couch which is separated by a curtain. Stick adhesive pads and disposable, sterile needles (gauge: 0.25 × 25 mm or 0.25 × 40 mm; Hwato, Suzhou, China)) will be used for acupuncture.

## 4 Acupuncture group

Participants in acupuncture group will receive therapy at the Lianquan (RN23), Renying (ST9), Danzhong (RN17), Guanyuan (RN4), Qihai (RN6), Zusanli (ST36), Sanyinjiao (SP6) and Zhaohai (KI6) points. The acupuncture points are identified according to the method of point location issued by the WHO. After local area disinfection with alcohol wipes, RN4, RN6, ST36, KI6 and SP6 will be inserted perpendicularly 10 to 15 mm deep with a 0.25 mm × 40 mm acupuncture needle. RN17 (with the needle tip pointing towards the head) will be punctured obliquely 5 to 10 mm deep with a 0.25 mm × 25 mm acupuncture needle, while RN23 (with the needle tip pointing towards the root of tongue) will be punctured obliquely to the depth of 5 mm. ST9 will be punctured perpendicularly to a depth of 5 mm. De qi sensation (a compositional sensation including numbness, soreness, distention, heaviness) will be achieved through lifting, thrusting, and rotating.

## 5 Sham acupuncture group

Participants in the control group will undergo treatment with a special sham acupuncture device with a blunt-tip needle (customize from Suzhou Huatu Medical Devices Co. Ltd.) which is similar to Streitberger needles device, without insertion.^[59]^ This non-invasive placebo needles can provoke a needling sensation as soon as it touches the skin. The acupoint selection and procedures will be the same as in the acupuncture group. The acupuncturists should pretend to manipulate the needle for 10 seconds on each point as well. No deqi sensation will be induced.

## 6 Outcome measures

### 6.1 Primary outcome

The primary outcome in this trial is the titers of TPOAb and TGAb at baseline and at the 4th, 8th, and 12th week after randomization.

### 6.2 Secondary outcomes

The secondary outcomes include:

(1) thyroid function test including free triiodothyronine (fT3), free thyroxine (fT4), and thyroid stimulating hormone (TSH);
(2) ovarian function including Anti Mullerian Hormone(AMH), follicle stimulating hormone (FSH), luteinizing hormone (LH), and estradiol (E2);
(3) pregnancy outcome including pregnancy rate (%), pregnancy losses (%), and live birth rate (%);
(4) primary ovarian insufficiency (%).

The thyroid function test will be performed at the baseline, 4th, 8th, and 12th week after randomization. The ovarian function will be examined on the 2nd to 4th day of the menstrual period in the 1st month, 2nd month and 3rd month compared with baseline (-1st month). Both the pregnancy outcome and the rate of primary ovarian insufficiency will be evaluated at 6 months and 1 year after treatment.

### 6.3 Assessment of adverse events

Any adverse events, including acupoint hematoma, bleeding, fainting, serious pain, infection, stuck needles, and broken needles, will be recorded by the researcher. If adverse events occur during the intervention, management will be taken emergently. Serious adverse reactions will be reported to the ethical committee and rescue procedures will be initiated at once.

### 6.4 Quality control and trial monitoring

Before the trial, all researchers involved will receive specialized training to maintain the study quality. The training courses include how to select and exclude patients, how to stratified randomization, how to manipulate interventions correctly, how to fill the case report form, how to assess outcomes and manage data. When the clinical trial begins, the principle researcher will supervise the trial. The licensed acupuncturists who have more than 5 years of experience will perform intervention according to the pre-specified standard operating procedure. Raw data will be recorded in the case record form (CRF). Two data managers enter the data into the spreadsheet and check the electronic data respectively. To guarantee the objectivity of the data, effect assessment and statistics will be blinded during the study period. Measures are taken to improve compliance, such as health education, respect patients informed consent right sufficiently etc.

### 6.5 Statistical analysis

Statistical analyses of data will be performed using SPSS21.0 software (International Business Machines Corp., Armonk, NY) by professionals who will be blinded to the whole trial process. According to the principle of intention-to-treat (ITT), all randomized participants will be analyzed. Continuous variables with normal distribution will be expressed as Mean ± Standard Deviation (M ± SD) and compared by an independent sample Student *t* test. For abnormally distributed variables, they will be expressed as Medians ± Interquartile Range (MIQR) and nonparametric tests will be used. Categorical variables will be presented by frequency and percentage and analyzed by χ^2^ test or Fisher exact test. A repeated-measures multifactorial analysis will be used to analyze value changes of TPOAb, TGAb, fT3, fT4, TSH, thyroxine, AMH, FSH, LH, and E2 across 4 testing time points (See Table 1). The rest of the secondary outcomes will compare the proportions of patients with primary ovarian insufficiency and different pregnancy outcome. Safety analyses will be compared with the incidence of AEs in two groups using the χ^2^ test. Missing data will be handled by multiple imputation methods. All the tests will be two-sided, and a *P* value of less than .05 will be considered statistically significant.

## 7 Discussion

Thyroid dysfunction and autoimmune in women of child-bearing age are adverse risk factors for fertility and pregnancy.^[36]^ The presence of anti-thyroid antibodies has an increased risk of unexplained infertility, low fertilization rates, poor embryo quality in assisted reproductive technologies, miscarriage, preterm delivery, perinatal mortality, and maternal post-partum thyroiditis.^[4,24]^ The relationship between reproductive failure and autoimmune conditions (including thyroid disease) has attracted worldwide attention in recent years.^[60]^ Consider that the early and middle stages of adulthood are periods of increased risk for many autoimmune diseases, particular emphasis was placed on the importance of reproductive problems in these groups.^[61]^ The association between TPOAb and subfertility has proposed several possible mechanisms^[21]^:

(1) The autoimmunity process may lead to subfertility or pregnancy loss;
(2) Infertility or pregnancy abortion may be secondary to hypothyroidism. An increased risk of unexplained infertility for the euthyroid women who were positive for thyroid antibodies.^[24]^

Prolonged mild hypothyroidism may have a negative impact on the ovarian follicular reserve.^[62]^ There is no specific treatment modality to suppress autoimmune destruction by modern medicine, therefore, seeking complementary and alternative therapy such as acupuncture in the early stage is of great significance for the treatment and fertility preservation in the child-bearing period female with HT.

Acupuncture therapy has been used in clinical practice on thyroid disease for a long time. Currently, there is a lack of high-quality research and evidence on acupuncture for Hashimoto's thyroiditis. Therefore, we are conducting a randomized controlled study to evaluate the effect of acupuncture on halting or delaying the progression of HT and fertility improving in the child-bearing period female. To reduce the possible bias, we use the stratified randomization method to randomize the enrolled patients into acupuncture group and sham acupuncture group. Due to the particularity of this invasive operation of acupuncture, completely inert placebo acupuncture and blinding is difficult in acupuncture trials. For the placebo control and better blinding of the participants, we use tailor-made sham needles and adhesive pads to treat participants alone in a separate room which is separated by a curtain. There is no definitively recommendation or objection to treatment with levothyroxine in euthyroid women who are positive for thyroid antibodies before pregnancy. In women seeking pregnancy through assisted reproductive technologies, the guidelines recommend levothyroxine treatment of subclinical hypothyroidism, defined as a TSH > 2.5 mIU/L in many studies, with a goal for the TSH of <2.5 mIU/L.^[50]^ Levothyroxine supplementation may improve fertility in infertile patients.^[63,64]^ Given that there may be some benefit and negative influence may be avoided with adequate levothyroxine therapy aimed at keeping TSH <2.5mIU/L, we will manage the participants whose TSH is greater than 2.5mIU/L with a typical starting dose LT4 (25 ug/d).

Thyroid peroxidase (TPO) and thyroglobulin(TG) are involved in thyroid autoimmunity as important antigens.^[65]^ TPO is the key enzyme in thyroid hormone synthesis.^[66]^ The thyroid hormones T3 and T4 are synthesized in the thyroid gland in a process that crucially involves the iodoglycoprotein thyroglobulin (TG).^[67]^ Antibodies against the main thyroid antigens (such as TPOAb and TGAb) can induce a chronic lymphocytic thyroiditis, eventually leading to the destruction and loss of thyroid function.^[21,68]^ TGAb may reflect a more initial type of immune response, while TPOAb can characterize later adaptive immune response, a sort of immune escalation. Positive TPOAb and/or positive TGAb, have been used test for screening for HT.^[69]^ TPOAb (positive in 95% of HT patients) as the best serological marker of HT is more sensitive than TGAb (positive in only 60%–80% of HT patients).^[7,70]^ Some surveys suggest that positive TPOAb are associated with an increased risk of developing hypothyroidism.^[11,12]^ In a cohort study, a TPOAb level above the threshold of 500 IU/ml was associated with a moderately increased risk for developing hypothyroidism.^[11]^ The presence of TPOAb negatively influences folliculogenesis, spermatogenesis, fertilization rates, embryo quality and pregnancy rates.^[71]^ In Chinese women, idiopathic low ovarian reserve is associated with more frequent positive TPOAb.^[32]^ Therefore, TPOAb and TGAb will be used as the primary outcome to assess the effect of acupuncture on delaying or preventing disease progression and improving fertility in child-bearing period female with HT. Except for thyroid antibodies, evaluation of thyroid function in patients with HT is carried out by measuring the serum levels of thyrotropin (TSH), free thyroxine (FT4), free triiodothyronine (FT3).^[7]^ Anti-Mullerian hormone (AMH) is secreted by the granulosa cells of growing ovarian follicles.^[72]^ AMH is a suitable biomarker of ovarian age in women of reproductive age and the most widely used and reliable serum biomarker of ovarian reserve.^[61,73]^ Follicle stimulating hormone (FSH) stimulates growth of ovarian follicles and aromatase expression in follicle granulosa cells. During reproductive aging in females, a rise in basal FSH is considered a sign of the reduction in the follicle reserve.^[74]^ Basal estradiol level is usually lower than 50pg/mL. It can indicate reproductive aging and hastened oocyte development if greater than 60 to 80 pg/mL in the early follicular phase.^[75]^ Luteinizing hormone (LH) plays an essential physiological role in follicle steroidogenesis and development and oocyte maturation. High basal LH levels are associated with significantly reduced fertilization rates and oocyte maturation, as well as impaired embryo quality, consequently resulting in an impaired pregnancy rate and higher miscarriage rate.^[76,77]^ Collectively, ovarian function will be assessed using the basal endocrine in the early follicular phase.

Acupoint selection has focused on Traditional Chinese medicine (TCM) theory or the treatment effects of acupuncture in this trial. SP6 and ST36 have been usually set for studies on endocrine disorder or reproductive syndrome.^[78,79]^ Electroacupuncture (EA) at the Guanyuan (RN4) or Sanyinjiao (SP6) can regulate hormone (E2, FSH, LH, GnRH) levels in the HPO axis.^[80]^ Acupuncture from Renying (ST9) to Shuitu (ST10) can regulate neuro-immune-endocrine system to return thyroid hormone levels to normal and reduce the titer of antithyroid antibody.^[81]^ EA at Guanyuan (RN4) and Zusanli (ST36) has benign regulating effects on the key hormones of pituitary-target gland axis,such as FSH, LH, E2, TSH, T3, and T4.^[55]^ Lianquan (RN23), Renying (ST9), Danzhong (RN17), Guanyuan (RN4) and Qihai (RN6) points are located in Ren meridian which runs along the anterior median line of body and means dominating pregnancy. The thyroid gland is closely related to the Ren meridian because it is located in the anterior neck. According to the basic principles of acupoint indications, acupuncture the points on Ren meridian can regulate the qi of Ren meridian to treat thyroid diseases and improve fertility. Lianquan (RN23) and Renying (ST9) in the anterior neck near the thyroid gland can regulate thyroid function directly. Zhaohai (KI6) as one acupoint of Foot-Shaoyin connects with Ren meridian to treat thyroid disease.

A limitation in this trial is that the acupuncturists cannot be blinded due to the nature of the intervention. We hope the results will provide reliable evidence and clarify the value of acupuncture as a treatment for child-bearing period female with HT.

## Author contributions

**Conceptualization:** Fangyuan Li, Zhang Qi, Lu Hua.

**Investigation:** Xinxin Wang.

**Supervision:** Mi Ling, Du Juan.

**Writing – original draft:** Fangyuan Li, Zhang Qi.

**Writing – review & editing:** Fangyuan Li, Lu Hua.
